# Supplementary material for: Phylogenetic conservation of bacterial responses to soil nitrogen addition across continents
Source: Nat Commun. 2019 Jun 7;10:2499. doi: 10.1038/s41467-019-10390-y (PMC6555827; doi:10.1038/s41467-019-10390-y)
Supplement: Supplementary file 1 — Supplementary information [file 41467_2019_10390_MOESM1_ESM.pdf]

Supplementary Information

Phylogenetic conservation of bacterial responses to soil nitrogen addition across continents

Isobe et al.

Supplementary Table 1. Sequences and OTUs of study sites included in the analysis

Locations	Sequence/metadata availability	Quality-filtered sequence count (average per plot)	Number of OTUs after removing the rare OTUs	Number of OTUs before removing the rare OTUs
Carey_1[1]	provided by the author(s)	318,762 (19,922)	1851	6058
Carey_2[1]	provided by the author(s)	341,450 (21,340)	2110	6444
Leff_1[2]	NCBI under ID PRJNA272747	262,811 (29,201)	2089	7231
Leff_2[2]	NCBI under ID PRJNA272747	168,264 (18,696)	1263	4398
Leff_3[2]	NCBI under ID PRJNA272747	170,314 (18,923)	1094	4795
Leff_4[2]	NCBI under ID PRJNA272747	141,589 (23,598)	1624	4210
Leff_5[2]	NCBI under ID PRJNA272747	208,601 (23,177)	1444	4799
Leff_6[2]	NCBI under ID PRJNA272747	141,463 (15,718)	1177	7127
Li_1[3]	provided by the author(s)	545,252 (45,437)	3303	9046
Li_2[3]	provided by the author(s)	577,412 (48,117)	2287	6327
McHugh[4]	MG-RAST under ID 4673309.3	321,687 (16,084)	1198	5106
OBrien[5]	provided by the author(s)	1,792,610 (298,768)	8612	12387
Wang[6]	ENA under ID PRJEB11700	170,167 (28,361)	2953	5481

Supplementary Table 2. *D* statistics of bacterial N response.

Locations	Positive response	Negative response
Carey_1[1]	**0.87**	**0.87**
Carey_2[1]	**0.86**	**0.86**
Leff_1[2]	**0.80**	**0.80**
Leff_2[2]	**0.83**	**0.83**
Leff_3[2]	**0.78**	**0.78**
Leff_4[2]	**0.93**	**0.93**
Leff_5[2]	**0.86**	**0.86**
Leff_6[2]	**0.88**	**0.87**
Li_1[3]	**0.55**	**0.55**
Li_2[3]	**0.68**	**0.68**
McHugh[4]	**0.76**	**0.77**
Obrien[5]	**0.91**	**0.91**
Wang[6]	**0.75**	**0.75**
Mean of each location	0.80	0.80
Merging locations	**0.78**	**0.78**

Bold indicates that the response is significantly associated with phylogeny (permutation test; $P < 0.05$).

Supplementary Table 3. Mantel correlation coefficient of branch lengths between OTUs in neighbor-joining and maximum likelihood trees (all $p < 0.001$), and the significance (p values) of positive and negative responses of the consenTRAIT algorithm (testing whether the response is significantly associated with phylogeny) using maximum likelihood trees (compare to Table 2). Italics indicates marginal significance.

Locations	Mantel coefficient	Positive response	Negative response
Carey_1[1]	**0.86**	*0.058*	0.279
Carey_2[1]	**0.87**	0.187	0.455
Leff_1[2]	**0.87**	**0.000**	0.827
Leff_2[2]	**0.84**	**0.003**	0.767
Leff_3[2]	**0.88**	**0.036**	0.398
Leff_4[2]	**0.87**	0.540	**0.072**
Leff_5[2]	**0.84**	**0.006**	0.491
Leff_6[2]	**0.90**	0.914	**0.002**
Li_1[3]	**0.82**	**0.000**	**0.000**
Li_2[3]	**0.82**	**0.015**	**0.000**
McHugh[4]	**0.86**	**0.008**	**0.002**
OBrien[5]	**0.80**	**0.005**	0.367
Wang[6]	**0.75**	**0.000**	**0.001**
Merging locations	**0.83**	**0.000**	0.578

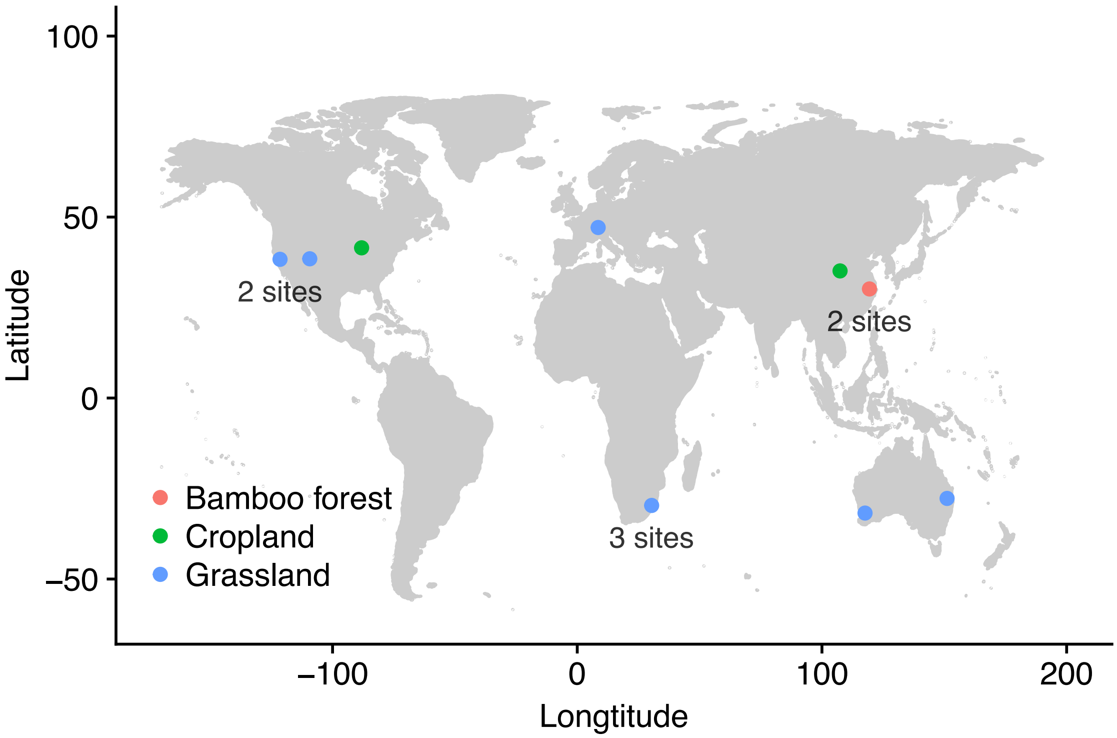

Supplementary Figure 1. Locations of N addition experiments. Some symbols overlap because they are in close proximity to one another.

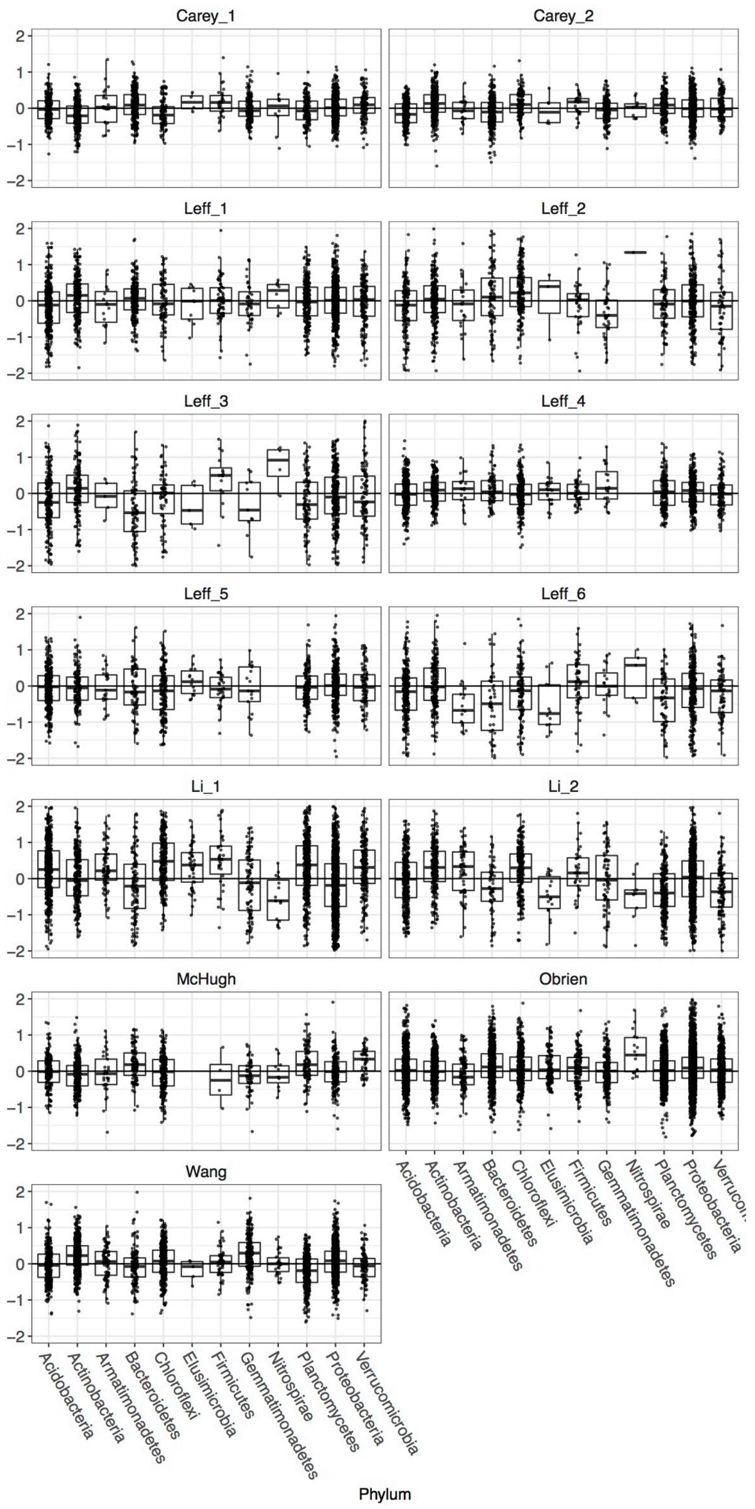

Supplementary Figure 2. OTU responses to N addition by site (each point), plotted by the most abundant phyla. The responses are calculated as a $\log_2$-fold ratio of averaged relative abundance in N addition plots relative to control plots. Note that the center line, box limits, and whiskers indicate the median, upper and lower quartiles, and 1.5x interquartile range, respectively.

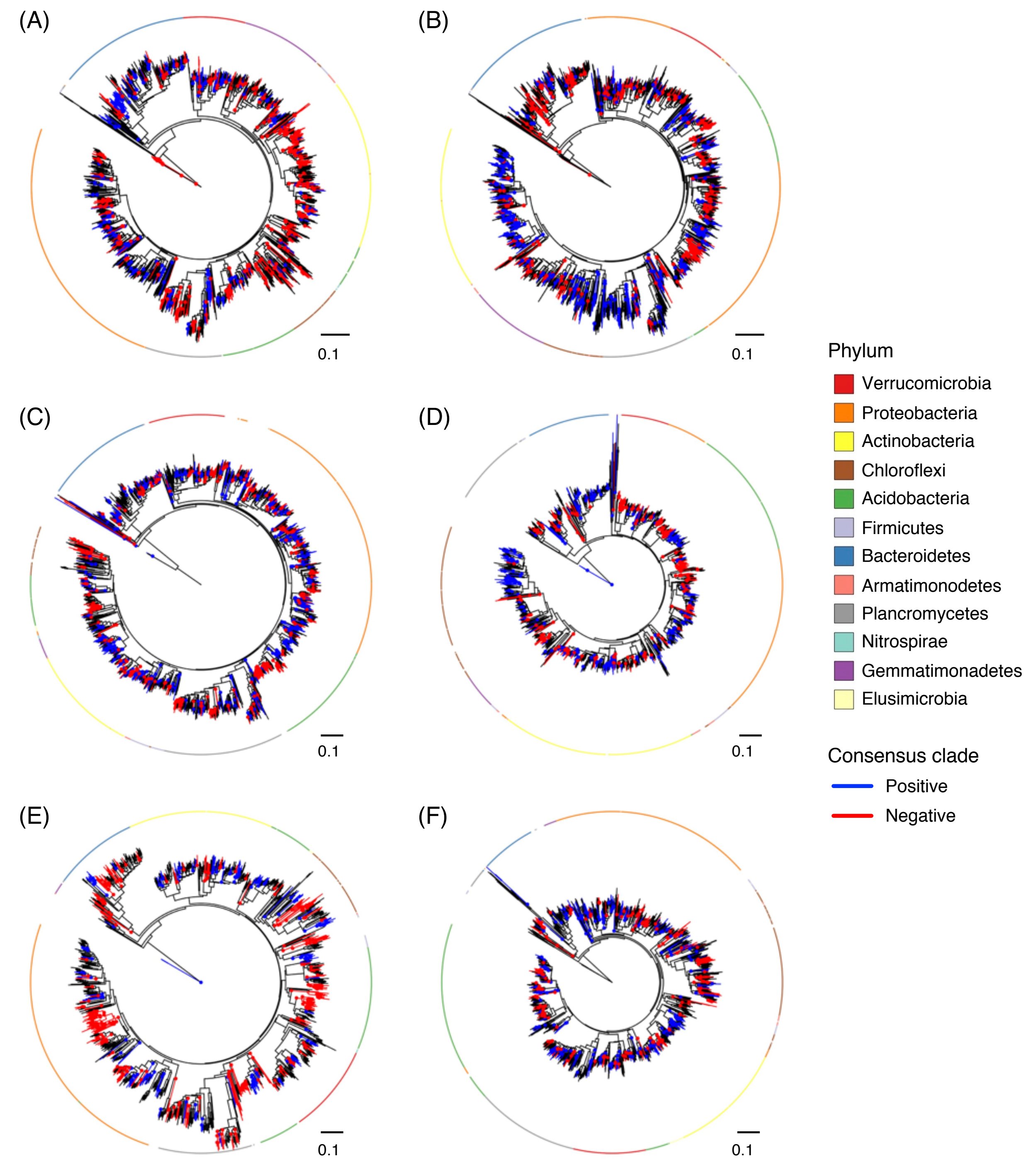

Supplementary Figure 3. Phylogenetic distribution of the response of bacterial OTUs to experimental N addition at each location; (A) Carey_1 (B) Carey_2 (C) Leff_1 (D) Leff_2 (E) Leff_3 (F) Leff_4 (G) Leff_5 (H) Leff_6 (I) Li_1 (J) Li_2 (K) McHugh (L) Obrien (M) Wang. Colored nodes and lineages show the consensus clades in which >90% of the descendant OTUs show the same direction of response (blue: positive response, red: negative response). The outer ring shows the phylum-level taxonomy of OTUs.

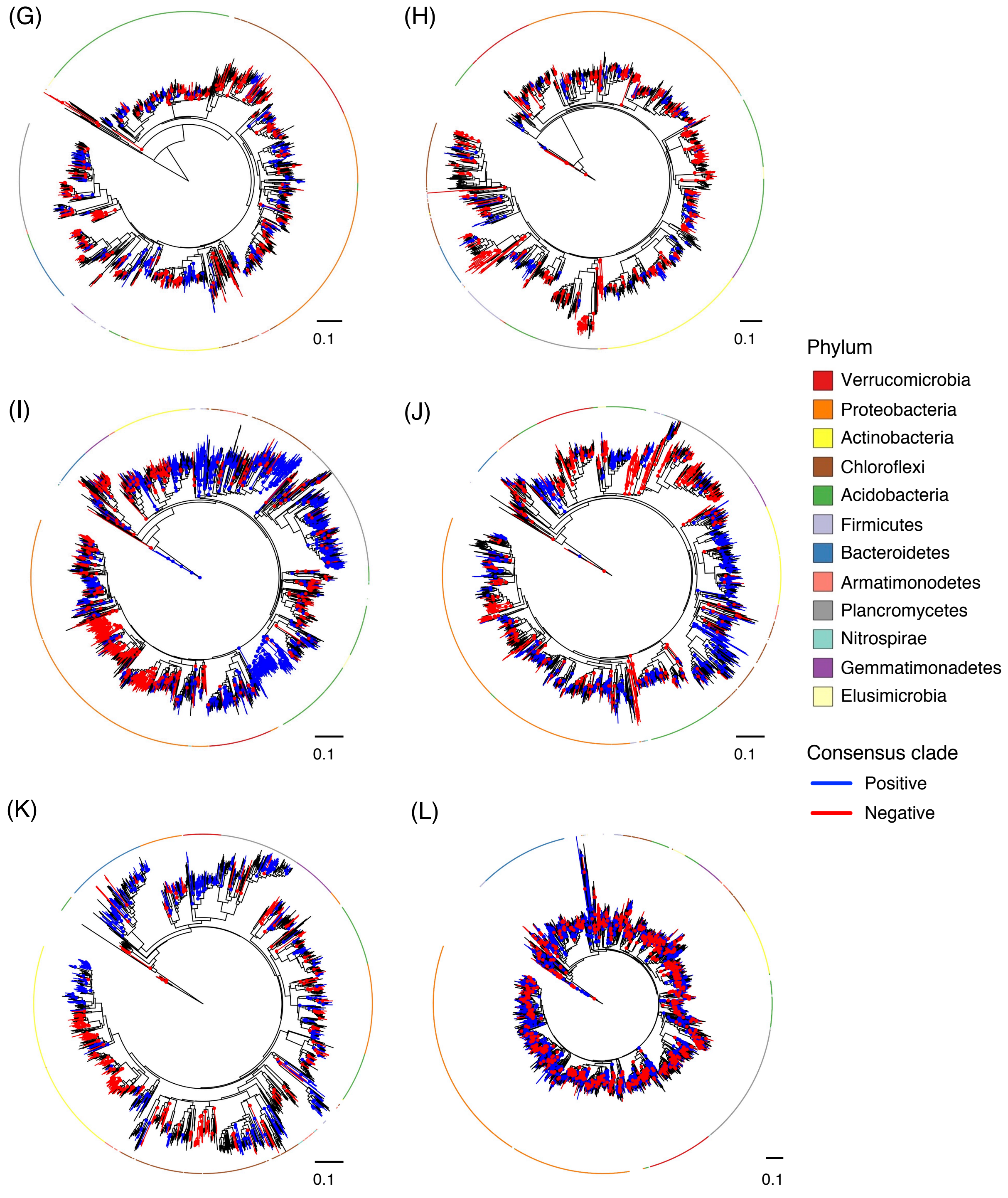

Supplementary Figure 3, continued.

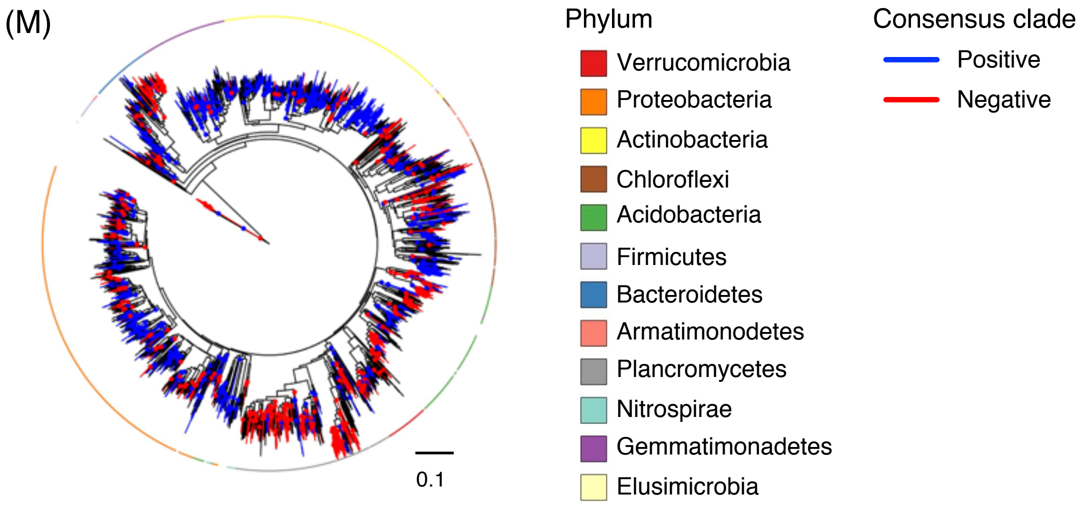

Supplementary Figure 3, continued.

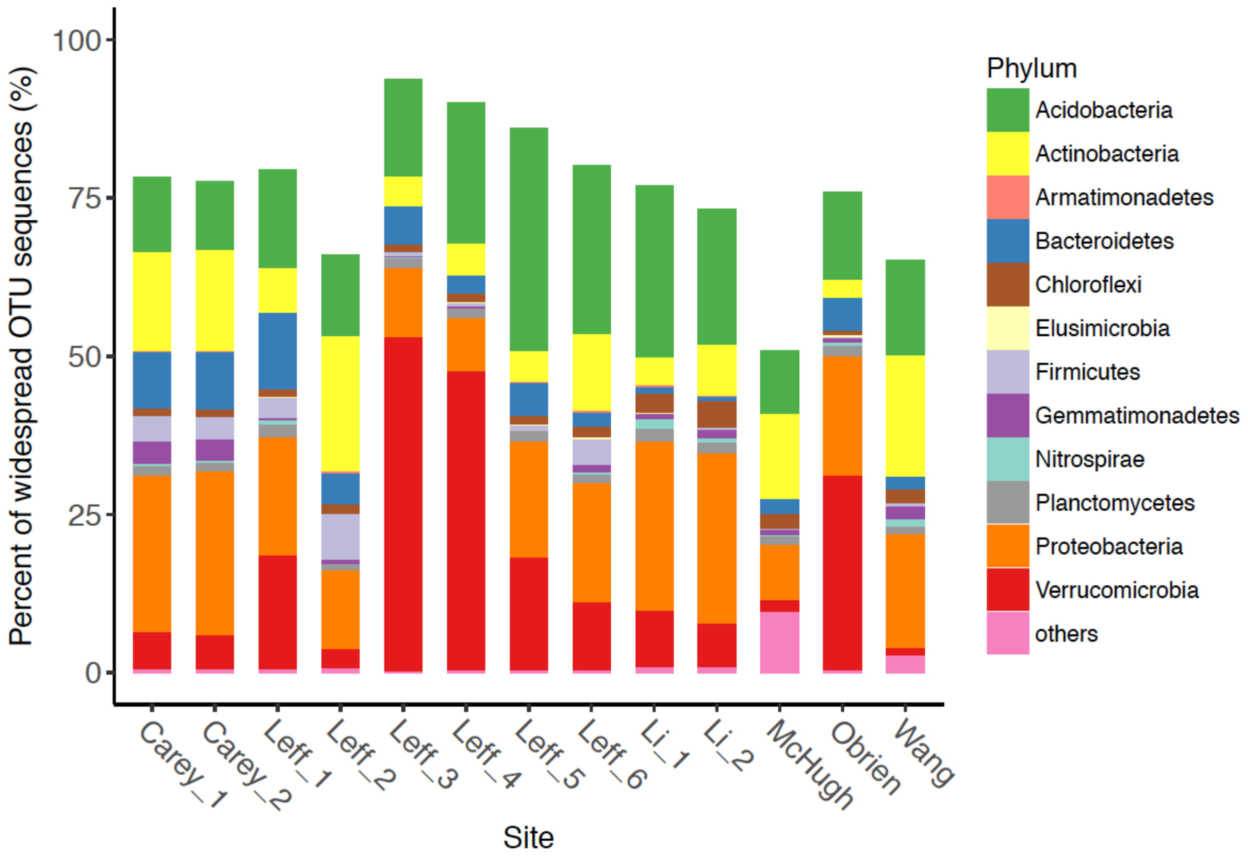

Supplementary Figure 4. Percent of widespread OTUs (i.e., present ≥5 locations) out of the total number of sequences from each location. Colors indicate the phylum of the widespread OTUs.

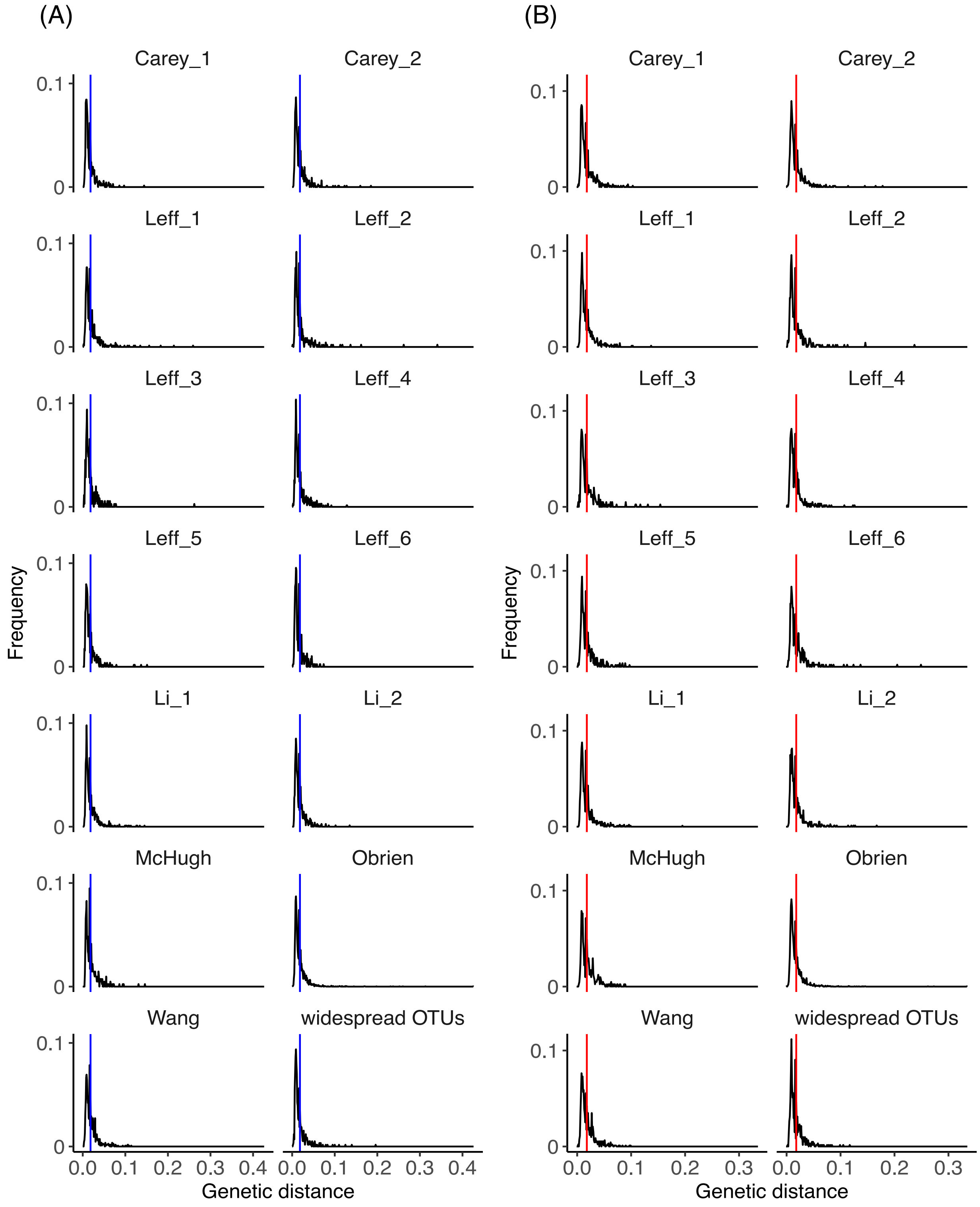

Supplementary Figure 5. Distributions of the genetic depth of consensus clades, those in which >90% of the descendant OTUs show the same direction of response; (A) positive and (B) negative responses. The vertical blue or red line shows the mean genetic depth.

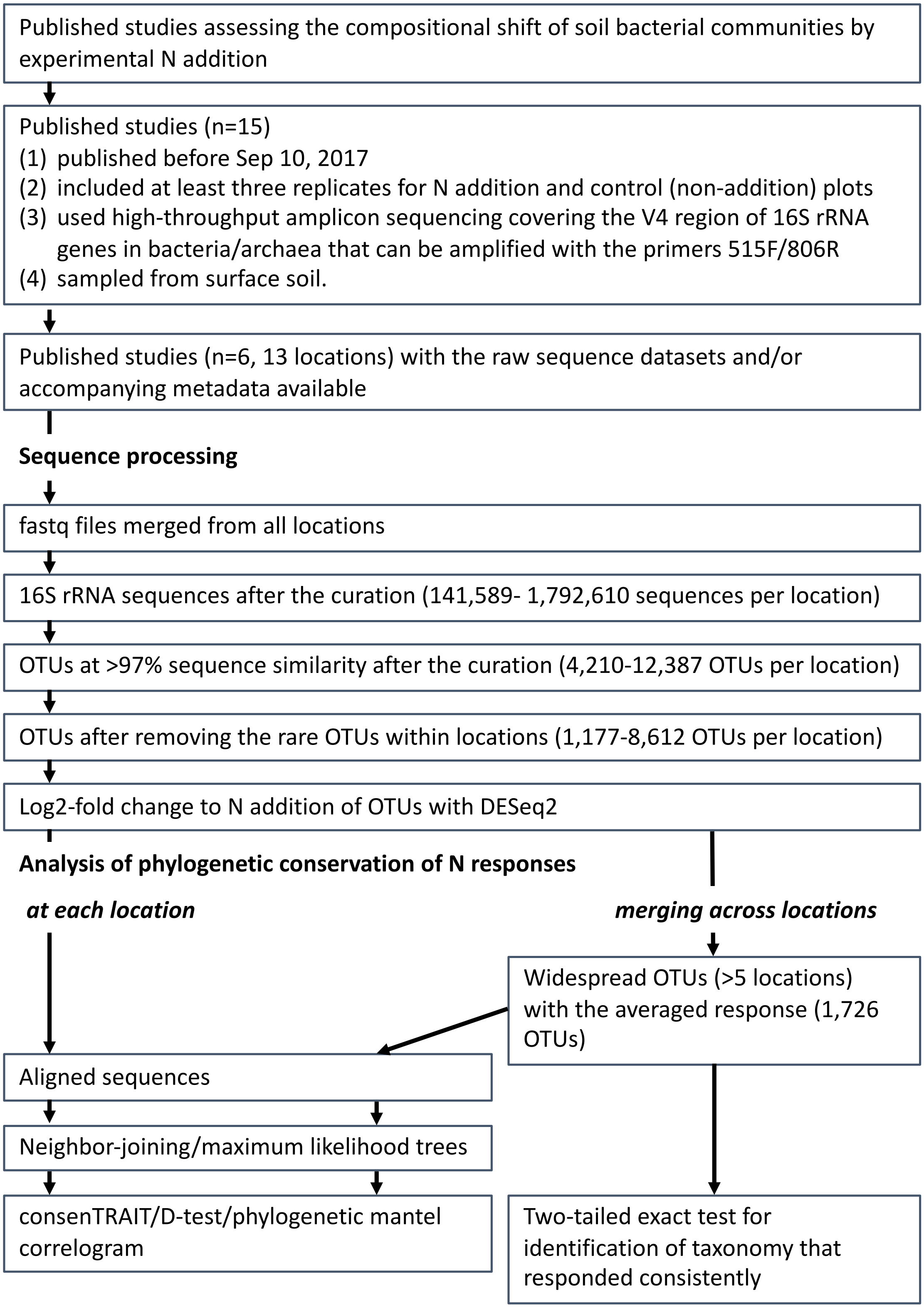

Supplementary Figure 6. Study inclusion criteria and flow diagram of methods.

Supplementary References

1. Carey, C. J., Beman, J. M., Eviner, V. T., Malmstrom, C. M. & Hart, S. C. Soil microbial community structure is unaltered by plant invasion, vegetation clipping, and nitrogen fertilization in experimental semi-arid grasslands. *Front. Microbiol.* **6,** (2015).
2. Leff, J. W. *et al.* Consistent responses of soil microbial communities to elevated nutrient inputs in grasslands across the globe. *Proc. Natl. Acad. Sci.* **112,** 10967–10972 (2015).
3. Li, Q., Song, X., Gu, H. & Gao, F. Nitrogen deposition and management practices increase soil microbial biomass carbon but decrease diversity in Moso bamboo plantations. *Sci. Rep.* **6,** 28235 (2016).
4. McHugh, T. A. *et al.* Bacterial, fungal, and plant communities exhibit no biomass or compositional response to two years of simulated nitrogen deposition in a semiarid grassland. *Environ. Microbiol.* **19,** 1600–1611 (2017).
5. O'Brien, S. L. *et al.* Spatial scale drives patterns in soil bacterial diversity. *Environ. Microbiol.* **18,** 2039–2051 (2016).
6. Wang, Y., Ji, H. & Gao, C. Differential responses of soil bacterial taxa to long-term P, N, and organic manure application. *J. Soils Sediments* **16,** 1046–1058 (2016).
