# Supplementary material for: Phylogenetic conservation of bacterial responses to soil nitrogen addition across continents
Source: Nat Commun. 2019 Jun 7;10:2499. doi: 10.1038/s41467-019-10390-y (PMC6555827; doi:10.1038/s41467-019-10390-y)
Supplement: Supplementary file 3 — Reporting Summary [file 41467_2019_10390_MOESM3_ESM.pdf]

nature research

Corresponding author(s): DBPR NCOMMS-18-34417A

Last updated by author(s): Apr 30, 2019

Reporting Summary

Nature Research wishes to improve the reproducibility of the work that we publish. This form provides structure for consistency and transparency in reporting. For further information on Nature Research policies, see Authors & Referees and the Editorial Policy Checklist.

Statistics

For all statistical analyses, confirm that the following items are present in the figure legend, table legend, main text, or Methods section.

n/a	Confirmed	
☐	☒	The exact sample size (n) for each experimental group/condition, given as a discrete number and unit of measurement
☐	☒	A statement on whether measurements were taken from distinct samples or whether the same sample was measured repeatedly
☐	☒	The statistical test(s) used AND whether they are one- or two-sided<br>*Only common tests should be described solely by name; describe more complex techniques in the Methods section.*
☒	☐	A description of all covariates tested
☒	☐	A description of any assumptions or corrections, such as tests of normality and adjustment for multiple comparisons
☒	☐	A full description of the statistical parameters including central tendency (e.g. means) or other basic estimates (e.g. regression coefficient) AND variation (e.g. standard deviation) or associated estimates of uncertainty (e.g. confidence intervals)
☒	☐	For null hypothesis testing, the test statistic (e.g. F, t, r) with confidence intervals, effect sizes, degrees of freedom and P value noted<br>*Give P values as exact values whenever suitable.*
☒	☐	For Bayesian analysis, information on the choice of priors and Markov chain Monte Carlo settings
☒	☐	For hierarchical and complex designs, identification of the appropriate level for tests and full reporting of outcomes
☒	☐	Estimates of effect sizes (e.g. Cohen's d, Pearson's r), indicating how they were calculated

Our web collection on statistics for biologists contains articles on many of the points above.

Software and code

Policy information about availability of computer code

Data collection	Not applicable
Data analysis	We used the open source softwares, UPARSE (usearch8.0.1517), QIIME (MacQiime 1.9.1) , and R (3.4.3).

For manuscripts utilizing custom algorithms or software that are central to the research but not yet described in published literature, software must be made available to editors/reviewers. We strongly encourage code deposition in a community repository (e.g. GitHub). See the Nature Research guidelines for submitting code & software for further information.

Data

Policy information about availability of data

All manuscripts must include a data availability statement. This statement should provide the following information, where applicable:

- Accession codes, unique identifiers, or web links for publicly available datasets
- A list of figures that have associated raw data
- A description of any restrictions on data availability

Raw sequence files and the associated metadata are available from the original studies, of which sources were summarized in Supplementary Table 1. Our reanalysis of the datasets is available at https://github.com/kazuo-isobe/phylogenetic-conservation-N-addition and from the authors upon request.

Field-specific reporting

Please select the one below that is the best fit for your research. If you are not sure, read the appropriate sections before making your selection.

☐ Life sciences ☐ Behavioural & social sciences ☒ Ecological, evolutionary & environmental sciences

Ecological, evolutionary & environmental sciences study design

All studies must disclose on these points even when the disclosure is negative.

Study description	We re-analyzed publicly available 16S rRNA gene data from soil N addition experiments and compared the bacterial communities between N addition and control plots.
Research sample	We used the 16S rRNA genes downloaded from the public database and provided by the authors.
Sampling strategy	We searched for published studies that assessed the compositional shift of soil bacterial/archaeal communities (based on 16S rRNA gene sequences) by the experimental manipulation of N addition that met the following criteria: (1) published before Sep 10, 2017, (2) included at least three replicates for N addition and control (non-addition) plots, (3) used high-throughput amplicon sequencing covering the V4 region of 16S rRNA genes in bacteria/archaea that can be amplified with the primers 515F/806R, and (4) sampled from surface soil.
Data collection	We downloaded the 16S rRNA gene from public database or asked the authors of original papers for the data.
Timing and spatial scale	We downloaded the data from the studies from 13 locations across 5 continents.
Data exclusions	No data were excluded.
Reproducibility	All attempts to repeat the experiment were successful.
Randomization	Due to the design and goals of our study, randomization was not necessary.
Blinding	Due to the design and goals of our study, blinding was not necessary.

Did the study involve field work? ☐ Yes ☒ No

Reporting for specific materials, systems and methods

We require information from authors about some types of materials, experimental systems and methods used in many studies. Here, indicate whether each material, system or method listed is relevant to your study. If you are not sure if a list item applies to your research, read the appropriate section before selecting a response.

Materials & experimental systems

n/a	Involved in the study
☒	☐ Antibodies
☒	☐ Eukaryotic cell lines
☒	☐ Palaeontology
☒	☐ Animals and other organisms
☒	☐ Human research participants
☒	☐ Clinical data

Methods

n/a	Involved in the study
☒	☐ ChIP-seq
☒	☐ Flow cytometry
☒	☐ MRI-based neuroimaging
